# Supplementary material for: A randomized phase 3 trial of Gemcitabine or Nab-paclitaxel combined with cisPlatin as first-line treatment in patients with metastatic triple-negative breast cancer
Source: Nat Commun. 2022 Jul 12;13:4025. doi: 10.1038/s41467-022-31704-7 (PMC9276725; doi:10.1038/s41467-022-31704-7)
Supplement: Supplementary file 1 — Supplementary Information [file 41467_2022_31704_MOESM1_ESM.pdf]

Contents **Page**

Contents	Page
Supplementary Note 1. Full List of GAP Study Investigators	2
Supplementary Table 1. Treatment exposure	4
Supplementary Table 2. Centrally assessed response to study treatment in ITT population	4
Supplementary Table 3. First-line regimens for metastatic triple-negative breast cancer from different studies	5
Supplementary Table 4. Number of patients with different response during the treatment.	6
Supplementary Table 5. Post-progression treatment	7
Supplementary Table 6. Distribution of treatment allocation and survival data in 9 centers	8
Supplementary References	9
Supplementary Note 2. Study Protocol	10

Supplementary Note 1. Full List of GAP Study Investigators

Investigator	Affiliation
Wenxia Peng	Fudan University Shanghai Cancer Center, Shanghai, China.
Leiping Wang	Fudan University Shanghai Cancer Center, Shanghai, China.
Jun Cao	Fudan University Shanghai Cancer Center, Shanghai, China.
Ting Li	Fudan University Shanghai Cancer Center, Shanghai, China.
Yiqun Du	Fudan University Shanghai Cancer Center, Shanghai, China.
Zhonghua Tao	Fudan University Shanghai Cancer Center, Shanghai, China.
Mingchuan Zhao	Fudan University Shanghai Cancer Center, Shanghai, China.
Sheng Zhang	Fudan University Shanghai Cancer Center, Shanghai, China.
Chengcheng Gong	Fudan University Shanghai Cancer Center, Shanghai, China.
Shuo Wu	Cancer Hospital of China Medical University, Liaoning Cancer Hospital and Institute, Shenyang, Liaoning, China.
Huan Li	Cancer Hospital of China Medical University, Liaoning Cancer Hospital and Institute, Shenyang, Liaoning, China.
Yanxia Shi	Sun Yat-Sen University Cancer Center, Guangzhou, Guangdong, China.
Zhongyu Yuan	Sun Yat-Sen University Cancer Center, Guangzhou, Guangdong, China.
Danni Li	The First Hospital of China Medical University, Shenyang, China.
Jing Shi	The First Hospital of China Medical University, Shenyang, China.
Pang Hui	Harbin Medical University Cancer Hospital, Harbin, China.
Xuesong Chen	Harbin Medical University Cancer Hospital, Harbin, China.
Mengwei Zhang	Henan Cancer Hospital, Zhengzhou, China.
Huimin Lv	Henan Cancer Hospital, Zhengzhou, China.
Zhanhong Chen	Zhejiang Cancer Hospital, Hangzhou, China.
Yabin Zheng	Zhejiang Cancer Hospital, Hangzhou, China.

Tao Wang	The Fifth Medical Center of Chinese PLA General Hospital, Beijing, China.
Huiqiang Zhang	The Fifth Medical Center of Chinese PLA General Hospital, Beijing, China.
Manping Chen	The First Hospital, Anhui Medical University, Hefei, China.
Xinghua Han	The First Hospital, Anhui Medical University, Hefei, China.

Supplementary Table 1. Treatment exposure

	Nab-paclitaxel plus cisplatin (n=127)	Gemcitabine plus cisplatin (n=126)
Cycles		
Total	849	698
Median (Range)	6(1-10)	6(0-10)
Relative dose intensity		
Cisplatin	23.7 mg/m^2 per week (22.3-24.2), 95%	23.5 mg/m^2 per week (21.5-24.5), 94%
Nab-paclitaxel/Gemcitabine	76.7 mg/m^2 per week (73.3-80.3), 92%	766.7 mg/m^2 per week (723.3-789.7), 92%

Data are median (IQR), %; IQR, interquartile range

Supplementary Table 2. Centrally assessed response to study treatment in ITT population

	ITT population		
Response	ABX+DDP (n=127)	GEM+DDP (n=126)	P-value
Complete response	12 (10.2%)	3 (2.4%)	
Partial response	89 (70.9%)	71 (56.3%)	
Stable disease	22 (15.7%)	37 (29.4%)	
Progressive disease	0 (0%)	7 (5.5%)	
Missing data or not assessable*	4 (3.1%)	8 (6.3%)	
Overall response	101 (79.5%)	74 (58.7%)	<0.001

*Tumor assessment data were missing or not assessable for response because of consent withdrawal before the first assessment in the ITT population.

P-value is given for chi-square test (2-sided) with no adjustment for multiple comparisons.

Supplementary Table 3. First-line regimens for metastatic triple-negative breast cancer from different studies

Trial	Regimen	No. of patients	ORR	Median PFS (month)	Median OS (month)
GAP	Nab-paclitaxle+cisplatin	127	81.1%	9.9	26.3
	Gemcitabine+cisplatin	127	55.9%	7.5	22.9
CBCSG006 [1]	Gemcitabine+cisplatin	118	64%	7.73	19.37
	Gemcitabine+paclitaxel	118	49%	6.07	18.07
IMpassion130 [2, 3]	Nab-paclitaxel+atezolizumab	451	56.0%	7.2	21
	PD-L1 positive	185	58.9%	7.5	25
	Nab-paclitaxel+placebo	451	45.9%	5.5	18.7
	PD-L1 positive	184	42.6%	5.3	18
Keynote355 [4]	Chemotherapy+pembrolizumab	566	NA	7.5	NR
	CPS＞10%	136	NA	9.7	NR
	Chemotherapy+placebo	281	NA	5.6	NR
	CPS＞10%	103	NA	5.6	NR
Miles et al. [5]	Chmotherapy+bevacizumab	363	NA	8.1	18.9
	Chmotherapy+placebo	258	NA	5.4	17.5

NA, not available; NR, not reached.

Supplementary Table 4. Number of patients with different response during the treatment.

Patients reached ORR (n/%)	2 cycles	4 cycles	6 cycles
AP	93 (73.2)	102 (80.3)	103 (81.1)
GP	63 (49.6)	69 (54.3)	71 (55.9)
Patients evaluated as PD (n/%)	2 cycles	4 cycles	6 cycles
AP	0 (0)	3 (2.4)	12 (9.4)
GP	7 (5.5)	11 (8.6)	29 (22.8)

Supplementary Table 5. Post-progression treatment.

Post-progression treatment	Nab-paclitaxel plus cisplatin (n=101)	Gemcitabine plus cisplatin (n=101)	P value
Combination regimens	32(31.7)	30(29.7)	0.76
Taxanes	5(5.0)	8(7.9)	0.27
Anthracycline	3(3.0)	2(2.0)	0.65
Vinorelbine	14(13.9)	17(16.8)	0.56
Capecitabine	7(6.9)	8(7.9)	0.79
Other fluoropyrimidine derivatives	2(2.0)	0(0)	0.48
Etoposide	0(0)	2(2.0)	0.48
Gemcitabine	5(5.0)	0(0)	0.07
Cyclophosphamide	3(3,0)	1(1.0)	0.61
Platinum	11(10.9)	6(5.9)	0.2
Bevacizumab	6(5.9)	5(5.0)	0.75
Apatinib	6(5.9)	7(6.9)	0.77
PD-1 antibody	1(1.0)	2(2.0)	1
Eribulin	0(0)	1(1.0)	NA
PARP inhibitor	1(1.0)	0(0)	NA
Single-agent regimens	32(31.7)	24(23.8)	0.21
Capecitabine	16(15.8)	9(8.9)	0.13
Vinorelbine	10(9.9)	12(11.9)	0.65
PARP inhibitor	4(4.0)	1(1.0)	0.36
Taxanes	1(1.0)	2(2.0)	1
Apatinib	1(1.0)	0(0)	NA
Radiotherapy for central nervous system metastasis	2(2.0)	2(2.0)	1
Local and regional therapy	5(5.0)	3(3.0)	0.72
Unknown	3(3.0)	5(5.0)	0.72

NA. not available.

P-value is given for chi-square test or Fisher exact test (2-sided) with no adjustment for multiple comparisons.

Supplementary Table 6. Distribution of treatment allocation and survival data in nine centers.

	Nab-paclitaxel plus cisplatin (n=127)	Gemcitabine plus cisplatin (n=126)	P value	PFS (95%CI)	OS(95%CI)
Fudan University Shanghai Cancer Center	75(59.1)	73(57.9)	0.73	9.4(8.2-10.5)	23.7(8.1-38.2)
Cancer Hospital of China Medical University	11(8.7)	16(12.7)		9.0(8.3-9.9)	20.0(13.6-27.5)
Sun Yat-Sen University Cancer Center	11(8.7)	13(10.3)		4.1(3.4-4.8)	NR
The First Hospital of China Medical University	8(6.3)	5(4.0)		13.0(2.4-17.7)	20.5(13.6-27.5)
Harbin Medical University Cancer Hospital	4(3.1)	7(5.6)		9.4(6.5-12.3)	12.1(3.8-20.4)
Henan Breast Cancer Center	7(5.5)	3(2.4)		10.3(3.5-24.7)	NR
Zhejiang Cancer Hospital	4(3.1)	5(4.0)		14.1(3.5-24.7)	36.3(21.0-51.7)
The Fifth Medical Center of Chinese PLA General Hospital	4(3.1)	3(2.4)		8.4(1.4-11.1)	30.0(1.6-58.4)
The First Hospital, Anhui Medical University	3(2.4)	1(0.8)		7.0(NR)	7.0(NR)

P-value is given for chi-square test or Fisher exact test (2-sided) with adjustments for multiple comparisons. NR. not reached.

Supplementary Reference

1. Hu XC, Zhang J, Xu BH, et al. Cisplatin plus gemcitabine versus paclitaxel plus gemcitabine as first-line therapy for metastatic triple-negative breast cancer (CBCSG006): a randomised, open-label, multicentre, phase 3 trial. Lancet Oncol 2015;16:436-46.
2. Schmid P, Adams S, Rugo H S, et al. Atezolizumab and Nab-Paclitaxel in Advanced Triple-Negative Breast Cancer[J]. N Engl J Med, 2018,379(22):2108-2121.
3. Schmid P, Rugo HS, Adams S, et al. Atezolizumab plus nab-paclitaxel as first-line treatment for unresectable, locally advanced or metastatic triple-negative breast cancer (IMpassion130): updated efficacy results from a randomised, double-blind, placebo-controlled, phase 3 trial. Lancet Oncol 2020;21:44-59.
4. Cortes J, Cescon D W, Rugo H S, et al. KEYNOTE-355: Randomized, double-blind, phase III study of pembrolizumab + chemotherapy versus placebo + chemotherapy for previously untreated locally recurrent inoperable or metastatic triple-negative breast cancer[J]. J Clin Oncol, 2020,suppl(38):1000.
5. Miles D W, Dieras V, Cortes J, et al. First-line bevacizumab in combination with chemotherapy for HER2-negative metastatic breast cancer: pooled and subgroup analyses of data from 2447 patients[J]. Ann Oncol, 2013,24(11):2773-2780.

Supplementary Note 2

A phase III, multicenter, randomized study of Abraxane plus cisPlatin versus Gemcitabine plus cisplatin (GAP) as first-line treatment in patients with metastatic triple-negative breast cancer

Version 1.2

Date 2016-03-24

No. CBCSG 018

Main site: Fudan University Shanghai Cancer Center

Principal investigator: Xichun Hu, Biyun Wang

Principal investigator:
Xichun Hu, Biyun Wang

Co-investigator and participating site:

Cancer Hospital Chinese Academy of Medical Sciences	Binghe Xu
	Ruigang Cai
Sun Yat-Sen University Cancer Center	Shusen Wang
Harbin Medical University Cancer Hospital	Li Cai
The First Hospital of China Medical University	Yue-E Teng
The First Hospital, Anhui Medical University	Yueyin Pan
Liaoning Cancer Hospital and Institute	Tao Sun
Hunan Cancer Hospital	Quchang Ouyang
The Fifth Medical Center of Chinese PLA General Hospital	Zefei Jiang

Protocol summary

Study design	A multi-center, randomized control phase III GAP clinical trial
Primary Endpoint	Progression Free Survival (PFS)
Secondary Endpoint	Objective Response Rate (ORR)<br>Safety<br>Overall Survival (OS)
Patient characteristic	First line treatment for TNBC
Treatment	Group A： AP<br>ABX 125 mg/m2 ivgtt 30 min d1, 8<br>DDP 75 mg/m2 ivgtt 120 min d1<br>Group B： GP<br>GEM 1250 mg/m2 ivgtt 30 mins d1, 8<br>DDP 75 mg/m2 ivgtt 120 mins d1<br>【Caution：Hydration should be applied when using DDP for 3 days to prevent renal toxicity】<br>Patients meeting inclusion criteria were randomized into AP or GP group.<br>Treatment repeated every 21 days until PD confirmed according to RECIST1.1 or intolerable toxicity.
Evaluation standard	RECIST 1.1
Adverse Events	CTCAE 4.0

1. Background

Breast Cancer is the most common cancer in women worldwide, which also ranks first in morbidity of Chinese women cancer [1, 2].With the development of breast cancer molecular subtypes, more and more drugs are applied for treatment, including chemotherapy, endocrine therapy, anti-HER2 treatment, etc. However, chemotherapy still plays an important role in (neo) adjuvant treatment, triple-negative breast cancer (TNBC), endocrine resistant breast cancer and patients with visceral crisis.

Triple-negative breast cancer (TNBC), defined as lacking estrogen receptor (ER) and progesterone-receptor (PgR) expression and amplification of human epidermal growth factor receptor 2 (HER2) gene, accounts for 12-17% of all breast cancers [3]. Patients with early TNBC are more likely to experience earlier relapse and shorter survival time, compared with other subtypes with a median disease free survival of 1-2 years and median survival time of 1-1.5 years for metastatic disease [4, 5].

Study shows TNBC shares similar molecular characteristics with basal-like breast cancer, with 70-90% consistency of molecular expression [6]. 11.2%-14.3% TNBC have BRCA1/2 mutation, which is closely related to homologous recombination deficiency [7, 8]. These provide a foundation for DNA cross-linking agents like platinum in the treatment of TNBC.

Much attention for platinum treatment has been aroused in early and advanced TNBCs and evidences suggest that platinum-containing regimens clinically benefit patients with TNBC. We previously conducted a phase III, multi-center clinical trial (CBCSG 006) during 2009-2014, comparing gemcitabine plus cisplatin (GP) and paclitaxel plus gemcitabine (GT) [9]. The results showed 7.73 months of PFS for GP group and 6.47 months for control group, indicating a favorable efficacy of GP to GT ($P_{superiority}$=0.009). In safety study, GP group showed higher rate of grade 3 or 4 nausea and vomiting, muscle and musculoskeletal pain, anemia, thrombocytopenia; GT group showed higher rate of grade 1-4 aplopecia and peripheral neurotoxicity. This study was the foundation of platinum-containing regimens in first-line therapy of mTNBC.

A phase II, prospective, single-arm study in our center of nab-paclitaxel (ABX) plus DDP in mTNBC [10]. 16/73 (22%) patients enrolled were TNBC, with ORR of 68.8%, a median PFS of 10.3 months and a median survival of 22.5 months. This study proved the efficacy and safety of nab-paclitaxel plus cisplatin in TNBC.

Several studies demonstrated that platinum-based treatment for TNBC is safe and efficient, and cisplatin-based treatment is standard treatment for metastatic TNBC. However, which drug is the best partner of cisplatin remains unknown. We considered nab-paclitaxel is the most sufficient single agent in metastatic breast cancer, thus, we designed this phase III, multi-center, randomized control clinical trial, aiming at comparing efficacy and safety of AP and GP in first-line treatment of TNBC and providing more evidences for clinical choice.

2. Endpoints

2.1 Primary Endpoint

Progression Free Survival, PFS

2.2 Secondary Endpoint

Objective Response Rate, ORR

Safety

Overall Survival, OS

3. Study Plan

3.1 Study design

This phase III, open-label, randomized clinical trial was conducted to compare nab-paclitaxel plus cisplatin and gemcitabine plus cisplatin group in PFS, ORR, OS and safety. Patients were randomized to receive nab-paclitaxel plus cisplatin or gemcitabine plus cisplatin and were evaluated every 2 treatments until progressive disease according to RECIST1.1 or intolerable toxicity.

3.2 Flow chart

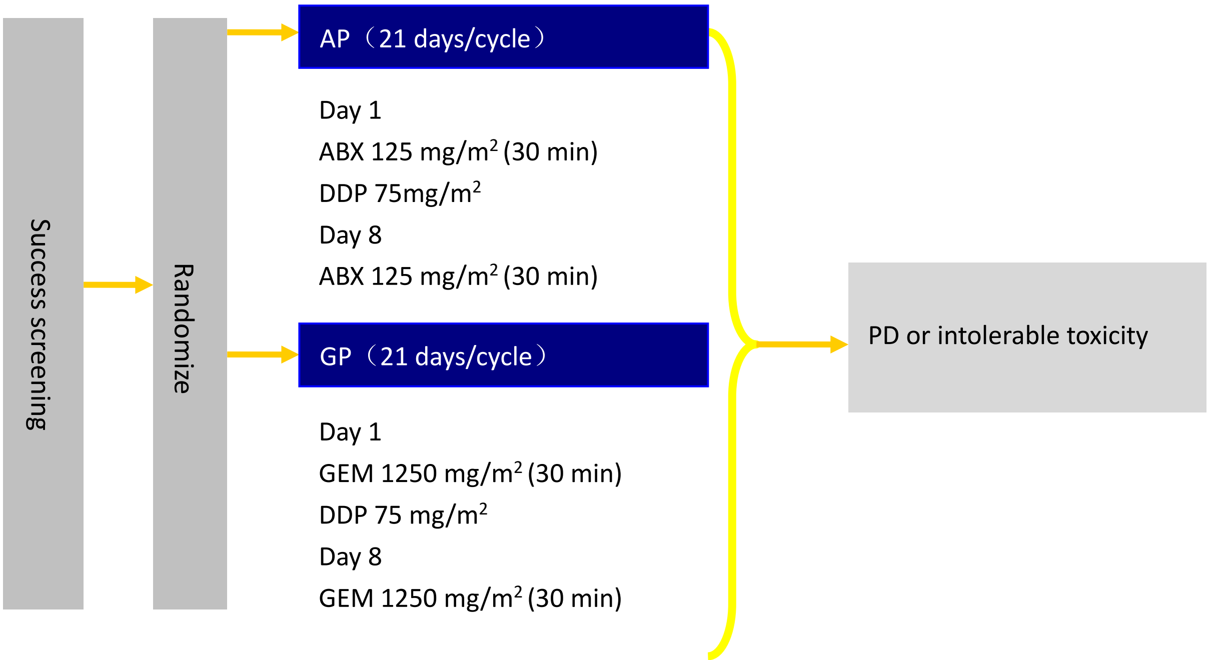

3.3 Randomization

Patients should be randomized to treatment group in this study to avoid bias. The inclusion and exclusion criteria are checked before randomization. Patients were randomized into AP or GP group using web-response system.

Stratification factors were the presence or absence of visceral metastasis (yes or no) and number of metastatic sites (1, 2 or ≥3). We use central dynamic randomization system to randomize patients into AP or GP group. 254 patients were randomized in this study, each group had 127 patients.

4. Patients

4.1 Inclusion Criteria

Patients should meet all inclusion criteria：

1. Female with age between 18 and 70 years old.
2. Histologically confirmed unresectable recurrent or advanced breast cancer, including de novo stage IV disease.
3. Estrogen receptor-negative (ER), progestogen receptor-negative (PR), and human epithelial receptor-2 (HER2)-negative by immunohistochemistry (ER <1%, PR <1% and HER2 negative). HER2 gene amplification should be verified by fluorescence in situ hybridization (FISH) test for those patients with HER2 (2+). For those with HER2 (1+), FISH test might be considered by the investigator.
4. No prior chemotherapy for metastatic breast cancer. Prior administration of chemotherapy in the adjuvant/neoadjuvant setting is acceptable with an interval ≥ 6 months before the enrollment.
5. At least one measurable disease according to RECIST 1.1.
6. ECOG ≤ 1.
7. All patients enrolled are required to have adequate hematologic, hepatic, and renal function.
8. Life expectancy longer than 12 weeks.
9. No serious medical history of heart, lung, liver and kidney.
10. Be able to understand the study procedures and sign informed consent.

4.2 Exclusion Criteria

Patients meet one of the following will be excluded：

1. Pregnant or lactating women (female patients of child-bearing potential must have a negative serum pregnancy test within 14 days before the first day of drug dosing, or, if positive, a pregnancy should be ruled out by ultrasound).
2. Women of child-bearing potential, unwilling to use adequate contraceptive protection during the process of the study.
3. Treatment with radiotherapy at the axial skeleton within 4 weeks before the first treatment or has not recovered from all toxicities of previous radiotherapy.
4. Treatment with an investigational product within 4 weeks before the first treatment
5. Patients with symptomatic central nervous system metastases are not permitted, except for those with stable and asymptomatic brain metastases who have completed cranial irradiation, and have at least one measurable lesion outside the brain. Radiotherapy should be completed within 4 weeks prior to the enrolment.

6. Other active malignancies (including other hematologic malignancies) or other malignancies within the last 5 years (patients with basal cell skin carcinoma and cervical carcinoma in situ were permitted).
7. Patients with any concurrent medical disorders which reduce hematological, hepatic, or renal, heart, endocrine, neurological or psychiatric functions.
8. Uncontrolled serious infection.

5. Treatment plan

Patients enrolled were randomized into AP or GP group.

Group A：AP

Nab-paclitaxel 125 mg/m^2，ivgtt，30 min，d1, 8

Cisplatin 75 mg/m^2，ivgtt，120 min，d1

【Caution：Hydration should be applied when using DDP for 3 days to prevent renal toxicity】

Group B：GP

Gemcitabine 1250 mg/m^2，ivgtt，30 min，d1, 8

Cisplatin 75 mg/m^2，ivgtt，120 min，d1

【Caution：Hydration should be applied when using DDP for 3 days to prevent renal toxicity】

Treatment repeated every 21 days and continued until PD according to RECIST1.1 or intolerable toxicity.

6. Supportive and Concurrent Drug

6.1 Preventive treatment

6.1.1 Antiemetic regimen

DDP had gastrointestinal toxicity with severe nausea and vomiting. Acute vomiting often appeared after 1-2 hours of DDP treatment and could last for one week. Standard antiemetic regimen was recommended including 5-HT antagonist, aprepitant and dexamethasone, day 1-3; or use promethazine instead, 25mg im, day 1-3 (for patients who refused to use aprepitant).

6.1.2 Hydration

DDP nephrotoxicity is mainly renal tubular damage. Acute damage is generally seen at 10 to 15 days after administration, which is characterized by blood urea nitrogen (BUN) and creatinine (Cr) increase, creatinine clearance rate decreases, and most of them are reversible. Repeated high-dose cisplatin can cause persistent mild to moderate renal damage. At present, there is no effective means to prevent nephrotoxicity caused by this drug except hydration. In this study, 75mg/m2 was used on the first day. Routine hydration treatment is recommended, and renal function should be monitored. You can refer to DDP instructions and hydration

principles for hydration treatment. Generally, it is recommended to hydrate for 3 days, with a daily intake of at least 2500ml. The amount of intravenous rehydration before and after the day of cisplatin is at least 2L, usually 1L before and after cisplatin (500ml of cisplatin itself is not included, but other fluids are included). Changes in renal tubular function and large amounts of fluid supplementation may cause electrolyte disturbances, and electrolyte disturbances can aggravate renal damage. Therefore, it is recommended to add potassium and magnesium electrolytes at the same time for hydration. According to literature reports, the general amount of potassium chloride is 20-30mmol/L, taking 2L of rehydration per day as an example, which is equivalent to 3-4.5g. It is recommended to supplement magnesium 40-80mmol per cycle of treatment, which is equivalent to add magnesium sulfate with 4.8-9.6g/cycle or adding magnesium chloride supplementation with 2.4-4.8g/cycle, generally after cisplatin. Studies have used magnesium sulfate 10mmol/L (1.2g) daily to add 1000ml of normal saline or magnesium sulfate 2-2.5g (16-20mmol/L) to 1000ml of normal saline. There is also a study showing that adding 8mmol of magnesium before cisplatin can reduce nephrotoxicity. The use of furosemide or mannitol is still controversial.

6.2 Supportive treatment

All patients can receive standard supportive care, including blood transfusion and platelet, antibiotics and antiemetic therapy.

6.2.1 Colony stimulating factor

Colony stimulating factors, such as G-CSF, can be used to treat chemotherapy-related neutropenia. Colony stimulating factor is not recommended as preventive treatment before treatment in this study. It is recommended for grade III/IV neutropenia and febrile neutropenia and is administered according to clinical routines.

6.2.2 Platelet transfusion and/or treatment of thrombocytopenia

For patients with grade 3/4 thrombocytopenia, interleukin-11 (IL-11), TPO, and/or platelet transfusion can be prescribed according to clinical routines.

6.2.3 Antibiotics

Some antibiotics (eg. aminoglycoside antibiotics, amphotericin B or cephalothin) increase nephrotoxicity when combined with DDP, which should be avoided during treatment. Other drugs can be used based on clinical principal.

6.2.4 Bisphosphonates and radiotherapy

Bisphosphonates are allowed as concomitant treatment, but should only be used when the renal function is normal. Pre-planned radiation therapy for bone metastasis is acceptable.

6.3 Concomitant treatment

In this study, concomitant treatment should be minimized. However, if concomitant treatment benefits the patients and doesn't have an effect on this study, the investigator can use it according to the specific situation, such as drugs to treat the concurrent chronic diseases. All concomitant treatments during the study (including 4 weeks before the initial administration) should be recorded in the medical record and ICF.

The following drugs are not recommended as concomitant medications of ABX, except for clinically necessary use:

Drugs that have the potential to induce CYP2C8 and CYP3A4, such as rifampicin, carbamazepine, phenytoin, efavirenz and nevirapine;

Drugs with potential inhibitory effects on CYP2C8 and CYP3A4, such as ketoconazole and other imidazole antifungal drugs, erythromycin, fluoxetine, gemfibrozil, cimetidine, ritonavir, saquinavir Indinavir and narfinavir.

The following drugs are not recommended as concomitant medications for DDP, except for clinically necessary use:

Aminoglycoside antibiotics, amphotericin B or cephalothin, etc. increase nephrotoxicity when combined with DDP, which should be avoided during treatment; methotrexate and bleomycin are mainly excreted by the kidneys, and the renal damage caused by treatment will delay the above the excretion of these drugs, that leads to increased toxicity. When probenecid is used in combination with treatment, it can cause hyperuricemia; chloramphenicol or its furanofenic acid or sodium diuretic can increase the ototoxicity of cisplatin; antihistamines can cover the toxicity of tinnitus, dizziness and other symptoms caused by cisplaitin.

7. Treatment delay and dose adjustment

Both ABX and GEM are administered intravenously for 30 min, on d1 and 8, in a 21 days cycle. Cisplatin are recommended to be administered after ABX and GEM.

7.1 General principles for dose modification

If ANC ≤ 1.5 x 10^9/L and platelets ≤ 100 x 10^9/L on d1 of each cycle, the treatment should be delayed. Patients with grade 3/4 peripheral neurotoxicity should discontinued the treatment, and the treatment will continue until neurotoxicity recovers to ≤grade 2. The treatment for patients with other ≤ grade 2 non-hematological toxicities were interrupted until the toxic effects resolved to a grade lower than 2 (except alopecia, nausea and vomiting). Unacceptable hematological or non-hematological toxicities occurred, the allocated treatment was delayed

for more than 14 days. Treatment was permanently discontinued if more than two dose modifi cations.

The dose reduction should be based on the most severe toxicity level of the previous cycle of treatment. Decrease the dose of chemotherapeutics by 20-25% for the first time and 20-25% for the second time, so that the dose can be reduced up to 50-60% of the initial dose. Those who is still not able to tolerate toxicity after two dose reductions will stop the treatment. Which drug should have dose-reduction depends on the toxicity of last cycle of treatment. For leucopenia or neutropenia, both of the two drugs should be reduced in two groups. For thrombocytopenia, only GEM should be reduced; when GEM-related pneumonia and hemolytic uremic occur, GEM is permanently stopped. Kidney toxicity, only adjust DDP.

7.1.1 Dose reduction of ABX, GEM and DDP

1. Febrile neutropenia (A disorder characterized by an ANC <1000/mm3 and a single temperature of >38.3 ℃ or a sustained temperature of ≥38 ℃ for more than one hour).
2. Grade 4 neutropenia sustained for ≥3 days.
3. For grade 3 peripheral neurotoxicity that resolve to ≤ grade 2 within 14 days, both nab-paclitaxel and cisplatin should be reduced in the AP group, while only cisplatin should be reduced in the GP group.

7.1.2 Dose reduction of GEM

1. Respiratory toxicity: Patients have ≥ grade2 pneumonitis, which is considered related to GEM, should discontinue GEM immediately.
2. Hemolytic uremic syndrome (HUS): Patients with HUS (A disorder characterized by a form of thrombotic microangiopathy with renal failure, hemolytic anemia, and severe thrombocytopenia) should discontinue GEM immediately.

7.1.3 Dose reduction of DDP

1. Renal toxicity

Modify the dosage of DDP according to renal function.

When 45ml/min≤creatinine clearance (CCI)<60ml/min, DDP is reduced by 25%

When 30ml/min≤creatinine clearance (CCI)<45ml/min, DDP is reduced by 50%

When creatinine clearance (CCI) is less than 30ml/min, DDP should be suspended. The treatment cannot be started until it resolve to ≤ grade 1.

7.2 The treatment of ABX and GEM on d8

Dose reduction or treatment delay is permitted for ABX and GEM on d8, which is determined by the investigator.

Dose reduction is based on the neutrophil and platelet counts on d8. When ANC≥1.2×10^9/L and PLT≥100×10^9/L on d8, the dose of ABX and GEM should not be reduced. The dose should be modified according to Table 1 when patients had a decreased neutrophil and/or platelet counts.

Treatment delay of ABX and GEM are also allowed within 7 days (that is d15), otherwise the treatment should be discontinued. If neutrophils don't recover to ≥1.2×10^9/L and platelets to ≥100×10^9/L after 7-day delay, the treatment on d8 of this cycle should be quitted.

Table 1: Dose modification of ABX and GEM on d8

Neutrophil count ($\times10^9$/L)		Platelet count ($\times10^9$/L)	Percentage of original dose
≥1.2	and	＞75	100
1.0-<1.2	or	50-75	75
0.7-<1.0	and	≥50	50
<0.7	or	<50	Suspended

8. Discontinuation of treatment and study

8.1 Discontinuation of treatment

Reasons for discontinuing a patient from this study are (follow-up should continue though the treatment is discontinued):

1. Disease progression (PD);

2. Intolerable toxicity;

3. Treatment delay for more than 2 weeks;

4. Pregnancy;

5. Other situations where the investigator considers it necessary to discontinue the treatment.

8.2 Discontinuation of study

The patient will discontinue the study in the following cases:

1. Withdrawal of informed consent;

2. Deterioration of concurrent diseases that will seriously affect the evaluation of efficacy or safety;

3. Treatment of other anti-tumor systemic treatments or the agents prohibited in this study;

4. Other situations where the investigator considers it necessary to discontinue the study.

9. Evaluation of patients

9.1 Baseline assessment

9.1.1 Medical history

Diagnosis, hormone receptor status and HER-2 receptor status, tumor size, lymph node metastasis, treatment history of breast cancer, menstrual status, concurrent diseases and concomitant medications, vital signs, ECOG and physical examination should be recorded.

9.1.2 Laboratory values

Blood routine, urine routine, liver and renal function, creatinine clearance rate etc. should be done within 1 week before enrollment. Tumor markers, electrocardiogram, etc. should be completed within 2 weeks before enrollment.

9.1.3 Tumor evaluation

A baseline assessment should be performed within 4 weeks before enrolment. Chest CT and abdominal MRI are recommended. Same examinations are required for the subsequent evaluation. Patients can receive bone scan and head CT or MRI at baseline when considered necessary. The RECIST 1.1 is used for tumor response evaluation.

9.2 Assessment during the treatment

9.2.1 Clinical evaluation

Vital signs, ECOG, and physical examination should be performed before next cycle of treatment.

9.2.2 Laboratory values

Blood routine, liver and renal function must be tested before next cycle of treatment, Next cycle of treatment can be continued after all the toxicity recover to ≤ grade 1.

9.2.3 Efficacy evaluation

Same examinations with baseline are required for the subsequent evaluation. Tumor assessment was performed at baseline and every two cycles until disease progression according to RECIST 1.1. If the patient discontinues treatment before the disease progresses, tumor assessment should be performed every 6 weeks until the disease progression.

The evaluation criteria of the objective response rate (ORR) refer to the RECIST 1.1: complete response (CR), partial response (PR), stable disease (SD) and progression of disease (PD). The evaluation of the target lesion (TL) progression should be compared with the time when the tumor burden is minimal (such as the minimum sum of the long diameters after the

start of the study). In addition to disease progression, other tumor remission indicators (CR, PR, and SD) were compared with baseline levels.

If the investigator is not sure whether the tumor is progressive, especially the increase of non-target lesions and the appearance of new lesions are equivocal, the treatment can be continued until the next evaluation, or a quick re-evaluation according to clinical needs. If repeat scans confirm there is definitely a new lesion or progression of non-target lesions, then progression should be declared using the date of the initial scan.

To achieve 'unequivocal progression' on the basis of the non-target disease, there must be an overall level of substantial worsening in non-target disease (even in presence of SD or PR in target disease), the overall tumor burden has increased sufficiently to merit discontinuation of therapy. A modest 'increase' in the size of one or more non-target lesions is usually not sufficient to quality for unequivocal progression status.

Before progressive disease, all patients who are receiving the treatments are evaluated every two cycles; those who discontinued the treatment are evaluated every 6 weeks. After progressive disease is reached, survival status is obtained every 3 months, and continue until death or loss to follow up. The evaluation should be carried out according to the assessment schedule, which can be referred to study design and Appendix 1.

The treatment of the patient (and the initial PFS evaluation) should be determined by the investigators according to RECIST assessment. However, it is necessary to collect all imaging assessment of the patients (including those in unspecified time and after progressive disease).

9.3 Safety analysis

Adverse events were evaluated at the end of each chemotherapy cycle, according to symptoms, signs or the abnormal results of an investigation (e.g., laboratory findings, electrocardiogram). Grade was assessed using National Cancer Institute Common Terminology Criteria for Adverse Events version 4.0.

10. Adverse event

10.1 Definition

An adverse event is the development of an undesirable medical condition or the deterioration of a pre-existing medical condition following or during exposure to the treatment, whether or not considered causally related to the treatment.

10.2 Adverse event monitoring

Adverse events were evaluated from the date of informed consent to 28 days after last dose of study drugs. Adverse events were recorded in the case report form (CRF), whether or not considered causally related to the treatment. Events, clearly the result of disease progression, should not be reported as an AE and death as a result of disease progression, should not be reported as an SAE.

10.3 Serious adverse event

A serious adverse event is an AE occurring during any study phase and fulfils one or more of the following criteria:

- results in death

- is immediately life-threatening

- requires in-patient hospitalisation or prolongation of existing hospitalisation

- results in persistent or significant disability or incapacity

- is a congenital abnormality or birth defect

- is an important medical event that may jeopardize the subject or may require medical intervention to prevent one of the outcomes listed above

However, other adverse events that may not be immediately life-threatening, result in death or hospitalization, may harm the patient or require immediate intervention to prevent the above results and are generally considered as serious adverse events. It is necessary to use a quick report system in the above situations.

10.4 Severity of Adverse Events

The investigators should evaluated the severity of adverse events and serious adverse events.

Severity is a measure of intensity whereas seriousness is defined by the criteria of serious adverse events. An AE of severe intensity need not necessarily be considered serious. For example, nausea that persists for several hours may be considered severe nausea, but not a SAE. On the other hand, a stroke that results in only a limited degree of disability may be considered a mild stroke but would be a SAE. The seriousness is based on the consequences or circumstances of the event, e.g. life-threatening or disability.

Adverse events are evaluated according to National Cancer Institute Common Terminology Criteria for Adverse Events (version 4.0) as grade 1-5. The adverse events that were not listed in CTCAE can be documented in the CRF based on the following criteria:

Mild: asymptomatic or mild symptoms; no limiting to activities of daily life; intervention not indicated.

Moderate: moderate symptoms; limiting instrumental activities of daily life; minimal, local or noninvasive intervention indicated;

Severe: serious symptoms; medical intervention indicated; hospitalization indicated; limiting self-care activities of daily life.

Life-threatening: life-threatening consequences; hospitalization and urgent intervention indicated.

Death.

10.5 Consequences

The relationship between the treatment and AE / SAE should be determined according to the following definitions, which can be assessed as not suspected or suspected:

Not suspected: A causal relationship between adverse events and study drug was observed according to the interval between administrations. Or other drugs, intervention or concurrent diseases provide sufficient explanation for the observed events.

Suspected: A high possibility of the relationship between adverse events and study drug according to the interval between administrations. Or other drugs, intervention or concurrent diseases cannot provide sufficient explanation for the observed events.

10.6 Reports of adverse events

The following variables will be collect for each AE and documented in the CRD: AE (verbatim); the date; when the AE started and stopped; intensity; whether the AE is serious or not; action taken with regard to the treatment; AE caused subject's withdrawal from study (yes or no); outcome. The investigator must inform GCP center and Ethics Committee in local centers of any SAE within 24 hours. Also, investigator must inform Celgene Drug Safety Site in China.

Celgene Drug Safety Site in China:

Telphone：+86 21 6133 9230/ +86 21 6133 9208

Fax：+86 400 630 7707

Email： drugsafety-china@celgene.com

11.Data and Study Management.

11.1Collection, monitoring, processing of data and archiving

Paper CRFs (pCRFs) and electronic CRFs (eCRFs) will be used to record study data required in protocol. Data should be recorded onto the pCRFs in blue or black ballpoint pen. Correction fluid or covering labels must not be used. The investigator will ensure that the data in the pCRFs are accurate, complete, and legible.

Data from the completed CRFs will be entered into clinical study database and validated under the direction of the Data Manager. Any missing, impossible, or inconsistent recordings in the CRFs will be referred back to the investigator.

AEs and medical history will be coded using the standard dictionary - Medical Dictionary for Regulatory Activities (MedDRA).

11.2Monitoring, Quality Control and Archiving of study

Before the first subject is recruited into the study, the local monitoring committee (MC) representative or delegate will:

- Establish the adequacy of the facilities and the investigator’s capability to appropriately select the sample
- Discuss with the investigator(s) (and other personnel involved with the study) their responsibilities with regards to protocol compliance.

During the study the local MC representative or delegate can implement different activities to assure compliance with standards of quality. These activities could include but are not limited to:

Contacts with the sites to:

Provide information and support to the investigator(s)

Confirm that the research team is complying with the protocol and that data are being accurately recorded in the case report forms (CRFs)

Ensure that the subject informed consent forms are signed and stored at the investigator's site

Ensure that the CRFs are completed properly and with adequate quality.

Monitoring activities for:

Checking a sample of ICFs

Checking that patients exist in medical records (a sample)

The extent and nature of monitoring will be decided during the study planning based on design, complexity, number of subjects, number of sites, etc.

If these, or any other signal occurs or if the local coordinator is suspicious of a potential non-optimal level of protocol compliance by the site investigator, specific measures should be adopted to evaluate the situation.

12. Sample size calculation

The primary endpoint of PFS was calculated for all intention-to-treat patients as the time from inclusion to disease progression or death from any cause. Group 1, nab-paclitaxel plus cisplatin, median PFS of 10.3 m; Group 2, gemcitabine plus cisplatin 7.73m. As first-line treatment for metastatic setting, nab-paclitaxel presented a median PFS of 12.8 and 12.9 months at the dose of 100 mg/m2 and 150 mg/m2 per week, respectively. We expected that AP improved the median PFS from 7.73 months of GP combination, based on our previous phase 3 study, to 11.5 months in patients with untreated mTNBC. The design had 80% power with a type 1 error of α=0.05 two-sided with phase III study. The statistical difference was considered significant if the P-value was less than 0.05. 115 patients were required to be enrolled each arm with the duration of 48 months and a follow up time of 12 months. The sample size was calculated as 127 for each group with a total of 254, including a 10% dropout rate.

13. Statistical analysis

13.1 Description of analysis set

Definition:

Safety set: all the patients who was treated with at least one dose of any study drug.

Intention-To-Treat population: all the patients who was treated with at least one dose of any study drug.

Long term follow-up set: all evaluable patients

Efficacy analyses were done on an intention-to-treat population. Safety analysis were based on Safety set. Other endpoints should be based on intention-to-treat population.

13.2 Methods of Statistical analysis

13.2.1 Patient characteristics

Descriptive statistics were used to analyze patient characteristics.

13.2.2 PFS

The primary endpoint was locally assessed progression free survival (PFS), defined as time from randomization to the first recorded occurrence of objective disease progression or death from any cause. Kaplan-Meier plots revealed median PFS and Log-rank tests were used to compare two treatment groups. Hazard ratio and corresponding 95% confidence intervals (CIs) were assessed by Cox proportional-hazard regression model. After treatment discontinue due to progression of disease, patients can receive subsequent treatments, and the follow-up is required according to the protocol.

13.2.3 OS

OS was defined as time from randomization to death due to any cause or the last visit of follow-ups. OS analysis was done on an intention-to-treat population. Kaplan-Meier plots revealed median PFS and Log-rank tests were used to compare two treatment groups. Patients who were not dead at follow-up were recorded as censored data and the endpoint was the last follow-ups.

13.2.4 ORR

ORR was defined as the percentage of patients who had a best objective tumor response of either complete response (CR) or partial response (PR). ORR was done on an intention-to-treat population. ORR was calculated by treatment group with 95% CIs, and compared with the Chi-square or Fisher exact test. After treatment discontinue due to progression of disease,

patients can receive subsequent treatments, but subsequent data will not be included in the evaluation of ORR.

13.2.5 Safety analysis

Safety analysis was done on a safety set. The summary of safety profile should include the number and percentage of patients with adverse events. The percentage of the patients is calculated according to the number of the patients in safety set.

14. Ethics and Informed Consent

14.1 Ethics

This study will be performed in accordance with ethical principles that are consistent with the Declaration of Helsinki, International Conference on Harmonization (ICH) /Good Clinical Practice (GCP) and ethical principles of Fudan University Shanghai Cancer Center.

14.2 Informed Consent

The Subject Informed Consent Form will incorporate wording that complies with relevant data protection and privacy legislation. The investigators are not allowed to give the genetic information to patients, insurance companies, employers, family members, doctors in charge and other third parties, except it is required by law.

14.3 Ethics review

The final protocol of the study, including the final version of the Subject Informed Consent Form, must be approved in writing by the Institutional Review Board/Independent Ethics Committee (IRB/IEC). The investigator will ensure the distribution of these documents to the IRB/IEC, and to the study site staff. The comments from IRB/IEC should have written records. The protocol can be reviewed annually by IRB / IEC when necessary according to the regulations of each site. The principal investigator is required to provide the reports of serious and unexpected adverse events to IRB/IEC. Genetic research should be incorporated in ICF and be approved by IRB/IEC, and writing records should also be reserved. The signed and dated subject informed consent must be obtained before enrolment.

14.4 Notes for informed consent process

The principal investigator should attention:

The investigator at each site will ensure that the subject is given full and adequate oral and written information about the nature, purpose, possible risk and benefit of the study.

Subjects must also be notified that they are free to discontinue from the study at any time.

The subjects should be given the opportunity to ask questions and allowed time to consider the information provided.

The signed and dated subject informed consent must be obtained before enrolment.

The Investigator must store the original, signed Subject Informed Consent Form.

A copy of the signed Subject Informed Consent Form must be given to the subject.

The Subject Informed Consent Form will incorporate wording that complies with relevant data protection and privacy legislation.

Possible risk and benefit of the study should be described in the ICF approved by the IEC.

Reference

[1] Siegel R, Naishadham D, Jemal A. Cancer statistics, 2012[J]. CA Cancer J Clin, 2012,62(1):10-29.

[2] Rongshou Z, Siwei Z, Liangyou W, et al. Incidence and mortality of malignant tumors in cancer registration regions of China in 2008 [J]. China Cancer, 2012, 21(1):1-12.

[3] Foulkes W D, Smith I E, Reis-Filho J S. Triple-negative breast cancer[J]. N Engl J Med, 2010,363(20):1938-1948.

[4] Dent R, Trudeau M, Pritchard K I, et al. Triple-negative breast cancer: clinical features and patterns of recurrence[J]. Clin Cancer Res, 2007,13(15 Pt 1):4429-4434.

[5] Kassam F, Enright K, Dent R, et al. Survival outcomes for patients with metastatic triple-negative breast cancer: implications for clinical practice and trial design[J]. Clin Breast Cancer, 2009,9(1):29-33.

[6] Gelmon K, Dent R, Mackey J R, et al. Targeting triple-negative breast cancer: optimising therapeutic outcomes[J]. Ann Oncol, 2012,23(9):2223-2234.

[7] Bergamaschi A, Kim Y H, Wang P, et al. Distinct patterns of DNA copy number alteration are associated with different clinicopathological features and gene-expression subtypes of breast cancer[J]. Genes Chromosomes Cancer, 2006,45(11):1033-1040.

[8] Andre F, Job B, Dessen P, et al. Molecular characterization of breast cancer with high-resolution oligonucleotide comparative genomic hybridization array[J]. Clin Cancer Res, 2009,15(2):441-451.

[9] Hu X C, Zhang J, Xu B H, et al. Cisplatin plus gemcitabine versus paclitaxel plus gemcitabine as first-line therapy for metastatic triple-negative breast cancer (CBCSG006): a randomised, open-label, multicentre, phase 3 trial[J]. Lancet Oncol, 2015,16(4):436-446.

[10] Sun S, Tang L, Zhang J, et al. Cisplatin improves antitumor activity of weekly nab-paclitaxel in patients with metastatic breast cancer[J]. Int J Nanomedicine, 2014,9:1443-1452.

Appendix 1. Study Plan

Study Plan	Screening（within 1 week before treatment）[1]	Every cycle（21days/cycle）		Every 2 cycles	End of the treatment	Follow-up
		D1	D8			
Written informed consent	×					Treatment until PD according to RECIST1.1 or intolerable toxicity. Survival status is obtained every 3 months, after PD is reached.
Inclusion/exclusion criteria	×					
Medical history	×			×	×	
Physical examination	×			×	×	
ECOG status	×			×	×	
Blood routine[2]	×	×	×			
Urine routine	×					
Liver and renal function[3]	×					
EKG	×					
Imaging examination[4]	×			×		
ABX or GEM		×	×			
DDP		×				
Concomitant drug	×	×	×	×	×	
Adverse event	×	×	×	×	×	

1. Treatment should be started within 1 week after randomization.
2. Blood routine should be done before every administration of chemotherapy, and additional tests can be added if necessary.
3. Liver and renal functions should be done within 1 week before every cycle of chemotherapy

(including total bilirubin, alanine aminotransferase, aspartate aminotransferase, alkaline phosphatase, blood urea nitrogen, blood creatinine), and additional tests can be added if necessary.

4. A baseline assessment should be performed within 4 weeks before enrolment. Chest CT and abdominal MRI are recommended. Same examinations are required for the subsequent evaluation. If the patient has no abdominal lesions, ultrasound examination can be done dependent on the investigators.
