# Supplementary material for: A randomized phase 3 trial of Gemcitabine or Nab-paclitaxel combined with cisPlatin as first-line treatment in patients with metastatic triple-negative breast cancer
Source: Nat Commun. 2022 Jul 12;13:4025. doi: 10.1038/s41467-022-31704-7 (PMC9276725; doi:10.1038/s41467-022-31704-7)
Supplement: Supplementary file 3 — Reporting Summary [file 41467_2022_31704_MOESM3_ESM.pdf]

nature portfolio

Corresponding author(s): Biyun Wang, Xichun Hu

Last updated by author(s): Jun 28, 2022

Reporting Summary

Nature Portfolio wishes to improve the reproducibility of the work that we publish. This form provides structure for consistency and transparency in reporting. For further information on Nature Portfolio policies, see our Editorial Policies and the Editorial Policy Checklist.

Statistics

For all statistical analyses, confirm that the following items are present in the figure legend, table legend, main text, or Methods section.

n/a	Confirmed	
☐	☒	The exact sample size (*n*) for each experimental group/condition, given as a discrete number and unit of measurement
☐	☒	A statement on whether measurements were taken from distinct samples or whether the same sample was measured repeatedly
☐	☒	The statistical test(s) used AND whether they are one- or two-sided<br>*Only common tests should be described solely by name; describe more complex techniques in the Methods section.*
☐	☒	A description of all covariates tested
☐	☒	A description of any assumptions or corrections, such as tests of normality and adjustment for multiple comparisons
☐	☒	A full description of the statistical parameters including central tendency (e.g. means) or other basic estimates (e.g. regression coefficient) AND variation (e.g. standard deviation) or associated estimates of uncertainty (e.g. confidence intervals)
☐	☒	For null hypothesis testing, the test statistic (e.g. *F*, *t*, *r*) with confidence intervals, effect sizes, degrees of freedom and *P* value noted<br>*Give P values as exact values whenever suitable.*
☒	☐	For Bayesian analysis, information on the choice of priors and Markov chain Monte Carlo settings
☒	☐	For hierarchical and complex designs, identification of the appropriate level for tests and full reporting of outcomes
☒	☐	Estimates of effect sizes (e.g. Cohen's *d*, Pearson's *r*), indicating how they were calculated

Our web collection on statistics for biologists contains articles on many of the points above.

Software and code

Policy information about availability of computer code

Data collection	No software was used.
Data analysis	All statistical analyses were carried out using SPSS software (version 19.0, SPSS Inc., Chicago, IL, USA).

For manuscripts utilizing custom algorithms or software that are central to the research but not yet described in published literature, software must be made available to editors and reviewers. We strongly encourage code deposition in a community repository (e.g. GitHub). See the Nature Portfolio guidelines for submitting code & software for further information.

Data

Policy information about availability of data

All manuscripts must include a data availability statement. This statement should provide the following information, where applicable:
- Accession codes, unique identifiers, or web links for publicly available datasets
- A description of any restrictions on data availability
- For clinical datasets or third party data, please ensure that the statement adheres to our policy

Data collected for the study, including individual participant data and a data dictionary defining each field in the set, will be made available to others; all available data will be deidentified participant data. The study protocol and statistical analysis plan are available (Supplementary Note 2). These data will be available with publication. The informed consent forms and ethics committee approval are available on a request submitted to Xichun Hu (xchu2009@hotmail.com) with a scientific proposal including objectives. These data are available under restricted access in compliance with patient consent and ethical principal for data sharing. The data will be shared after approval by Xichun Hu and by the investigators of the GAP trial within two weeks.

Field-specific reporting

Please select the one below that is the best fit for your research. If you are not sure, read the appropriate sections before making your selection.

- [x] Life sciences
- [ ] Behavioural & social sciences
- [ ] Ecological, evolutionary & environmental sciences

For a reference copy of the document with all sections, see nature.com/documents/nr-reporting-summary-flat.pdf

Life sciences study design

All studies must disclose on these points even when the disclosure is negative.

Sample size	Based on our previous phase 2 trial on AP, we observed a median PFS of 10.3 months in the mTNBC subgroup. We expected that AP improved the median PFS from 7.73 months of GP combination, based on our previous phase 3 study, to 11.5 months in patients with untreated mTNBC. A total of 115 patients were required with 80% power using a log-rank (Lakatos) test at a two-sided 5% level of significance, with a 4-year accrual, 1 year follow-up. Considering a 10% dropout rate and 1:1 randomization, a total of 254 patients were required, with 127 patients per group. The primary analysis was carried out on an intention-to-treat (ITT) population, defined as all the patients who was treated with at least one dose of any study drug. Safety analysis was performed in the same population.
Data exclusions	Key exclusion criteria were as follows: Patient of childbearing potential but unwilling to receive contraception, radiation therapy of axial bone within 4 weeks before enrollment in the study, treatment with an experimental agent within the previous 4 weeks, symptomatic central nervous system (CNS) disease, other malignant diseases within the past 5 years (patients with basal cell skin carcinoma and cervical carcinoma in situ were allowed), and uncontrolled infection.
Replication	Clinical data were collected in a paper-CRF by two individual investigators and the study was monitored according to ICH-GCP guidelines. Data extraction and statistical analysis of clinical outcomes were analysed by a professional statistician, Jianfeng Luo.
Randomization	Patients were randomly assigned in a 1:1 ratio to receive AP or GP. Randomization was carried out centrally with the use of a block randomization of size eight and an interactive web-response system. The stratification factors included visceral metastasis (yes or no) and the number of metastatic sites (1, 2, or ≥3). The investigators or research coordinators checked the inclusion and exclusion criteria and sent the random assignment forms by fax to the Clinical Research Coordination Office in Fudan University Shanghai Cancer Centre (Shanghai, China). The allocated treatment was sent back to the investigator by fax.
Blinding	This is a open-label trial because all the treatments in our study are approved chemical drugs and the aim of our study is to identify the anti-tumor effects of the combination. Investigators or patients can easily know what medicines the patients have taken according to the adverse events of the treatment.

Reporting for specific materials, systems and methods

We require information from authors about some types of materials, experimental systems and methods used in many studies. Here, indicate whether each material, system or method listed is relevant to your study. If you are not sure if a list item applies to your research, read the appropriate section before selecting a response.

Materials & experimental systems

n/a	Involved in the study	
x		Antibodies
x		Eukaryotic cell lines
x		Palaeontology and archaeology
x		Animals and other organisms
	x	Human research participants
	x	Clinical data
x		Dual use research of concern

Methods

n/a	Involved in the study	
x		ChIP-seq
x		Flow cytometry
x		MRI-based neuroimaging

Human research participants

Policy information about studies involving human research participants

Population characteristics	The median age was 50 (range 22–69) and 52 (range 30–75) in nab-paclitaxel+cisplatin (AP) and gemcitabine+cisplatin (GP) group, respectively. Most patients (85.8% in AP and 82.5% in GP) had an ECOG score of 1. Nearly half of the patients were in premenopausal status in both groups. Most patients experienced the recurrence at more than 1 year after breast operation. Lung metastasis was the most common metastatic site both in AP and GP groups (44.9% and 44.4%, respectively). Anthracycline and taxane in neoadjuvant or adjuvant setting were observed in 59.0% patients of AP group and 59.5% of GP group.
Recruitment	Investigators from 9 hospitals in China checked the outpatients who visited their clinic for the inclusion and exclusion criteria and then invited them to review the informed consent form. After the approval of informed consent form, the participants at different centers were screened for stratification factors to ensure the cohorts were balanced. The investigators then sent the random assignment forms by fax to the Clinical Research Coordination Office in Fudan University Shanghai Cancer Centre (Shanghai, China). The allocated treatment was sent back to the investigator by fax. The investigators gave the allocated treatment to participants at different centers.
Ethics oversight	The study protocol, statistical analysis plan and informed consent form were approved by ethics committee of Fudan University Shanghai Cancer Center.

Note that full information on the approval of the study protocol must also be provided in the manuscript.

Clinical data

Policy information about clinical studies

All manuscripts should comply with the ICMJEguidelines for publication of clinical research and a completedCONSORT checklist must be included with all submissions.

Clinical trial registration	NCT02546934
Study protocol	Study protocol was attached as supplementary materials in submission.
Data collection	Data collection was conducted by investigators by paper-CRF. Baseline data from 9 centers were collected between Mar 2015 and Oct 2019. Follow-up data were collected from 9 centers from Oct 2019 and Feb 2021.
Outcomes	The primary endpoint was locally assessed PFS, defined as the time from randomization to the first recorded occurrence of objective disease progression according to RECIST 1.1 or death from any cause. The secondary endpoints included objective response rate (ORR), overall survival (OS), and safety profile. AEs were graded using National Cancer Institute Common Terminology Criteria for Adverse Events version 4.0. The correlation between the AEs/serious AEs (SAEs) and the treatment was determined by the investigators. Any AE leading to the dose reduction, dose delay, dose miss or discontinuation was also recorded.
